# On Sanguineous Perspiration

**Published:** 1849-06

**Authors:** 


### PATHOLOGY AND PRACTICE OF MEDICINE.

*On Sanguineous Perspiration.* By Dr. SCHNEIDER.—It has often been a question whether, under any circumstances, blood is ever mixed with the fluid of perspiration in human beings. Dr. Schneider remarks that he has several times observed the phenomenon. He mentions having been once summoned to a healthy man, 50 years of age, who, for a period of 12 hours in succession, had travelled on foot; during the journey he had perspired much in his feet; and, on examining them at the end of it, they were found covered as high as the ankles with a sanguineous perspiration, which had also soaked into and stained his stockings. In another case of a healthy young man, Dr. S. mentions having noticed that, after violent exercise, the perspiration beneath the arms was of a bright red colour; and he quotes a similar case from Huffman.

In proof that the perspiration over the whole body may also be of a sanguineous character, he mentions one case in which it had been observed in a delicate man after copulation, and then quotes the following still more remarkable case from Paulini. While surgeon on board a vessel, a violent storm arose, and threatened immediate destruction to all. One of the sailors, a healthy Dane, 30 years of age, of fair complexion and light hair, was so terrified that he fell speechless on the deck. On going to him Paulini observed large drops of perspiration of a bright red colour on his face. At first he imagined the blood came from the nose, or that the man had injured himself by falling; but, on wiping off the red drops from the face, he was astonished to see fresh ones start up in their place. This coloured perspiration oozed out from different parts of the forehead, cheeks, and chin; but it was not confined to these parts, for, on opening his dress, he found it formed on the neck and chest. On wiping and carefully examining the skin, he distinctly observed the red fluid exuding from the orifices of the sudoriparous ducts. So deeply stained was the fluid, that on taking hold of the handkerchief with which it was wiped off, the fingers were made quite bloody. As the bloody perspiration ceased, the man's speech returned; and when the storm had passed over he recovered, and remained quite well during the rest of the voyage.—*Lond. Med. Gaz. from Casper's Wochenschrift.*

